# Advances in laboratory detection of acute kidney injury

**DOI:** 10.1016/j.plabm.2022.e00283

**Published:** 2022-06-02

**Authors:** Faeq Husain-Syed, Thiago Reis, Kianoush Kashani, Claudio Ronco

**Affiliations:** a Department of Internal Medicine II, University Hospital Giessen and Marburg, Justus-Liebig-University Giessen, Klinikstraße 33, 35392, Giessen, Germany; b Laboratory of Molecular Pharmacology, Faculty of Health Sciences, University of Brasília, Brasília, Distrito Federal, Brazil; c Department of Nephrology and Kidney Transplantation, Clínica de Doenças Renais de Brasília, DF Star Hospital, Rede D'Or São Luiz, Brasília, Distrito Federal, Brazil; d Division of Nephrology and Hypertension, Division of Pulmonary and Critical Care Medicine, Department of Medicine, Mayo Clinic, Rochester, MN, USA; e Department of Medicine (DIMED), Università di Padova, Via Giustiniani, 2–35128, Padua, Italy; f International Renal Research Institute of Vicenza, Via Rodolfi, 37–36100, Vicenza, Italy; g Department of Nephrology, Dialysis and Transplantation, San Bortolo Hospital, Via Rodolfi, 37–36100, Vicenza, Italy

**Keywords:** Acute kidney stress, Cell cycle arrest biomarkers, Subclinical AKI, Tubular damage

## Abstract

Recent advances have improved our understanding of the epidemiology and pathophysiology of acute kidney injury (AKI). So far, the Kidney Disease: Improving Global Outcome guidelines define and stratify kidney injury based on increases in serum creatinine level and/or decreases in urine output. Although the term AKI acknowledges the existence of cellular injury, its diagnosis is still only defined by the reduced excretory function of the kidney. New biomarkers that aid a better understanding of the relationship between acute tubular injury and kidney dysfunction have been identified, reflecting the advances in molecular biology. The expression of some of these novel biomarkers precedes changes in conventional biomarkers or can increase their predictive power. Therefore, they might enhance the clinical accuracy of the definition of AKI. This review summarizes the limitations of the current AKI classification and a panel of candidate biomarkers for augmenting AKI classification and recognition of AKI subphenotypes. We expect that the integration of appropriately selected biomarkers in routine clinical practice can improve AKI care.

## Author contributions

FH-S, TR, and KK prepared all manuscript drafts and were involved in reviewing and editing. CR was involved in the writing and editing of the manuscript, including the figures. In addition, CR conceived the concept underlying the manuscript, was involved in reviewing and editing, and is the paper's senior author.

## 1 Introduction

Over the last decade, there has been considerable progress in understanding acute kidney injury (AKI) epidemiology and pathophysiology and its diagnostic testing. AKI is now recognized as a collection of entities [1], including cardiorenal [2], hepatorenal [3], nephrotoxic [4], cardiac surgery-associated [5], sepsis-associated AKI [6], and AKI due to urinary tract obstruction. The evolution from a description of a single disease to specific syndromes has emerged as necessary given that these syndromes have distinct pathophysiologies and treatments. Furthermore, the identification and validation of several novel AKI biomarkers in different clinical settings have shown that they precede changes in established biomarkers and might increase their predictive power. Therefore, they might enhance the clinical accuracy of the definition of AKI and possibly guide therapy. This review summarizes recent advances in the conceptual framework and evolving terminology of AKI and the knowledge of novel AKI biomarkers, including their potential clinical implications.

## 2 Epidemiology of acute kidney injury

AKI epidemiology and etiology differ between high-vs. vs. middle-to low-income countries [7] and are also dependent on the population to be considered. In high-income countries, AKI occurs in 10–15% of hospitalized patients [8] and in 20–25% of patients who have undergone cardiac surgery [9]. AKI occurs in up to 60% of intensive care unit (ICU) patients [10]. In this setting, AKI-related costs are high, and prevention is difficult. Notably, AKI incidence in ICUs has increased over the past decades in world regions with aging populations [11,12]. In contrast, in the middle-to low-income countries, AKI is predominantly community-acquired and largely preventable, with dehydration being the most common cause [12,13]. AKI is considered a syndrome of multifactorial origin. Certain risk factors (e.g., age >65 years, chronic kidney disease [CKD], heart failure, diabetes mellitus) predispose to AKI development [14]. In developed countries, sepsis [10], major surgeries (e.g., cardiac surgery [9] and non-cardiac major surgery [10]), cardiorenal syndrome [15], and the use of nephrotoxic drugs [4] are common triggers of AKI. The complexity of AKI is evident from the fact that each syndrome has a unique pathophysiology and treatment.

Independent of its etiology, AKI is associated with increased short- and long-term mortality [16,17] and longer hospital stays [18]. The observed increased morbidity and mortality is partly due to the fact that AKI increases the risk of developing subsequent CKD [19] and is associated with heightened cardiovascular risk [20]. On the other hand, CKD and/or pre-existing cardiovascular disease predispose to AKI and increased morbidity and mortality [20].

## 3 Limitations of the current acute kidney injury classification

**Fig. 1:**
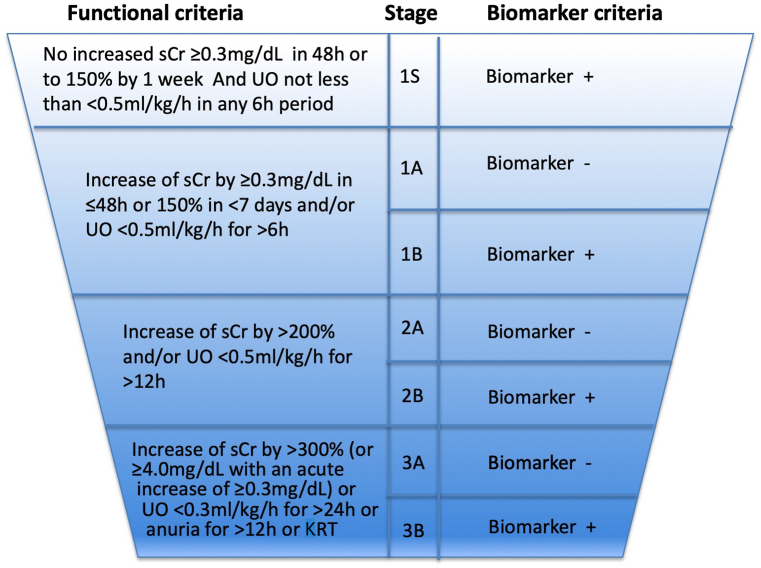
Current and proposed definition and staging of acute kidney injury. According to the 2012 KDIGO guidelines, current markers of AKI include sCr and/or UO for indicating kidney dysfunction (left panel). A combination of novel damage and functional markers, along with clinical information, might be used for identifying high-risk patients, improving AKI diagnostic accuracy and processes of care, and assisting AKI management (right panel). It should be noted that biomarker positivity should be based on its mechanism and defined threshold, which still requires validation. To convert sCr to mmol/l, multiply by 88.4.ADQI, Acute Dialysis Quality Initiative; KDIGO, Kidney Disease: Improving Global Outcomes; KRT, kidney replacement therapy; sCr, serum creatinine; UO, urinary output. ADQI Initiative 23. www.adqi.org. Used with permission.

AKI diagnosis and classification is based on the 2012 Kidney Disease: Improving Global Outcomes (KDIGO) consensus criteria [21] (Fig. 1, left panel). The 3-stage severity classification system relies on clinical assessment and evaluation of the absolute and relative changes in serum creatinine concentration based on a known or assumed baseline value or the decrease in urinary output over seven days [21]. Although the relationship between the KDIGO-based criteria for AKI diagnosis and staging and mortality and morbidity has been well documented [7], they have clear and important limitations that should be addressed. As such, serum creatinine and urinary output are surrogate markers of the glomerular filtration rate (GFR) and are not specific for AKI. GFR as measured by serum creatinine may not decrease until ≥50% of the functional nephrons are lost in previous healthy kidneys [22]. In addition, the serum creatinine concentration is affected by multiple confounders, such as muscle mass, volume status, or tubular handling. Furthermore, factors such as malnutrition and sarcopenia [21,23] and volume overload [24] may falsely indicate normal serum creatinine values, although AKI is already present. Similarly, renin-angiotensin-aldosterone system inhibitors or other drugs that affect the GFR may result in small changes in serum creatinine levels that are not indicative of kidney injury [25].

Compared to serum creatinine, urine output can be considered a more sensitive but less specific criterion for AKI [26]. Some forms of AKI primarily manifest with oliguria (e.g., in acute cardiorenal syndrome [27]). Notably, as serum creatinine and urinary output measure the loss of kidney function and not injury, AKI can be deemed a misnomer. In the absence of injury markers, individuals with episodes of volume depletion can meet the diagnostic criteria of AKI without injury being present. On the other hand, diuretic use may mask a relevant decrease in urinary output and delay AKI recognition.

Further limitations of the KDIGO-based criteria are that neither serum creatinine nor urinary output is suitable markers for the timely detection of AKI [23,28]. After an abrupt decrease in GFR in AKI, serum creatinine rises until a new equilibrium is reached between production and elimination [29]. Consequently, AKI is often diagnosed up to 2 days after the initial insult, which may take even longer in the context of volume overload [23,24].

## 4 Proposed new definition of acute kidney injury

**Fig. 2:**
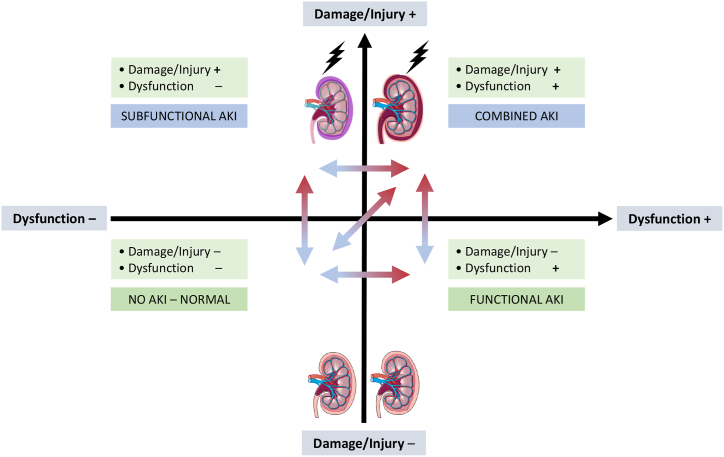
Conceptual framework of acute kidney injury syndromes based on functional and damage criteria. Modified from De Oliveira et al. [34]. The combination of damage/injury, functional kidney markers and clinical information may enrich AKI classification. Patients may be categorized as having i) no AKI (bottom left), ii) kidney damage/injury, subfunctional AKI (top left, defined as AKI stage 1S), iii) kidney dysfunction, functional AKI (bottom right, defined as AKI stage 1A, 2A, or 3A), iv) and a combination of kidney damage/injury and kidney dysfunction, or combined AKI (top right, defined as AKI stage 1B, 2B, or 3B). Red arrows show progression, whereas blue arrows show regression or resolution. Examples of functional AKI may include use of angiotensin-converting-enzyme inhibitors, angiotensin receptor blockers or sodium-glucose cotransporter-2 inhibitors, and conditions such as volume depletion, which can reduce GFR without damaging the kidney. AKI stages 1S, 1A–3A and 1B–3B were proposed by Ostermann et al. [32]. AKI, acute kidney injury; GFR, glomerular filtration rate.

Although decreased GFR characterizes AKI, the histopathologic correlate for AKI is often captured as acute tubular damage, which reflects intrinsic kidney damage as a result of either ischemic or toxic insult affecting the functional and structural integrity of renal tubules [28,30]. As existing technology cannot directly assess kidney damage, apart from biopsy, numerous urinary biomarkers are used or proposed as glomerular or tubular cell injury indicators to potentially enhance the clinical accuracy of the definition of AKI and guide therapy (Fig. 1, right panel) [31,32]. Conceptually, the spectrum of AKI ranges from subclinical AKI [33], a state indicating kidney damage biomarker positivity with no loss of function (i.e., KDIGO AKI criteria-negative), to decreased GFR without evidence of tubular damage, to manifest AKI with decreased GFR and increased kidney biomarkers [34] (Fig. 2).

Notably, subclinical AKI can be considered a misnomer, as AKI may not be longer “subclinical” when novel biomarkers can detect damage. However, AKI might still be “subfunctional”, as the presence of novel biomarkers might precede changes in dysfunction markers [35]. Therefore, novel kidney biomarkers are expected to predict and detect AKI in a timelier manner, allowing earlier initiation of preventive measures before a “functional” diagnosis of AKI can be made. Whether and if so, what consequences can be drawn in the event of a positive damage signal remains the subject of research. Studies indicate that early implementation of care bundles in biomarker-positive selected patients at high risk for cardiac surgery-associated AKI may reduce the rate of AKI stage 3 [36,37].

New kidney biomarkers have been extensively investigated in AKI [32]. Based on their specificity, they could be used for evaluating AKI etiology and severity and the site of injury [34]. Nevertheless, it remains to be clarified which novel kidney biomarker should be used depending on the etiology and temporal course of kidney damage, as they reflect diverse physiological and pathophysiological processes in the damaged tissue [38]. Furthermore, in most countries, the costs for implementing new kidney biomarkers in clinical practice are not reimbursed, so they are mainly used in research. Therefore, the approach for a patient with AKI depends on the clinical context and varies by resource availability. Integration of the new renal biomarkers into the KDIGO guidelines for AKI is currently being discussed [39].

## 5 Application of new acute kidney injury biomarkers

The discovery of AKI biomarkers has the potential to improve the AKI diagnostic approach and treatment substantially. Several molecules have been identified as potential markers for early detection of kidney damage before serum creatinine rises.

An ideal AKI biomarker reflecting acute tubular injury should have the following properties [40]: i) easily measured; ii) upregulated shortly after the injurious stimulus and downregulated after the termination of the stimulation; iii) a dose-dependent response, i.e., the biomarker quantity should be proportional to AKI severity; iv) of tubular origin; v) able to distinguish the injury induced by different types of AKI; vi) reflect the injury process, i.e., injury vs. repair; vii) its analysis should be complementary to the analysis of a “functional” biomarker. However, limitations in specificity (especially in patients with comorbid conditions), poor predictive performance when the timing of the kidney insult is unknown, and in some cases, suboptimal sensitivity have led to damage markers being used mainly for research purposes. Table 1 presents some new and established kidney biomarkers.

**Table 1:** Characteristics of acute kidney injury biomarkers.

Biomarker	Sample	Class	Origin	Molecular weight (kDa)
TIMP-2	Urine	Stress	Distal tubule [41]	21
IGFBP7	Urine	Stress	Proximal tubule [41]	25
NGAL	Urine or plasma	Damage	Distal tubule, epithelial cells throughout the body, neutrophils [32,42]	Three different types (25, 45, and 135)
KIM-1	Urine	Damage	Proximal tubule [43]	38.7
L-FABP	Urine	Damage	Proximal tubule [44]	14
CCL14	Urine	Damage	Multiple cell types throughout the body [45,46]	7.8
Dickkopf-3	Urine	Stress	Tubular epithelia [47]	38
Cystatin C	Plasma	Function	Nucleated cells throughout the body [48]	13.3
Proenkephalin A	Plasma	Function	Produced in the central nervous system, heart, kidney, intestine, lung, skeletal muscle, and immune cells [49]	4.5

Research is needed to establish which biomarkers will be qualified for augmenting AKI classification. Furthermore, it remains unclear when these biomarkers should be measured and what cutoffs are needed, and will require further investigation.

CCL14, C–C motif chemokine ligand 14; KIM-1, kidney injury molecule 1; L-FABP, liver-type fatty acid-binding protein; NGAL, neutrophil gelatinase-associated lipocalin.

### 5.1 Tissue inhibitor of metalloproteinase 2 and insulin-like growth factor-binding protein 7

The two urinary biomarkers TIMP-2 (tissue inhibitor of metalloproteinase 2; 21 kDa) and IGFBP7 (insulin-like growth factor-binding protein 7; 25 kDa) are secreted in the early phase of tubular damage (e.g., in the context of sepsis [50] or after ischemia [41]) by the proximal and distal tubular epithelial cells. In contrast to tubular damage markers (e.g., neutrophil gelatinase-associated lipocalin [NGAL] [32]), TIMP-2 and IGFBP7 can be released in response to non-injurious, noxious stimuli [41]. Both biomarkers are pre-formed in the tubular epithelial cells, so their expression does not require transcription [51]. For this reason, both biomarkers are also referred to as kidney stress markers [52]. Both biomarkers have been proposed as diagnostic tools for predicting AKI, diagnosing AKI, and estimating AKI severity [32]. Based on experimental work with various cell types, it is assumed that TIMP-2 and IGFBP7 block the effects of the cyclin-dependent kinases in an autocrine and paracrine manner during AKI, thereby inducing G1 cell cycle arrest of the tubular epithelial cells to prevent the division of cells with damaged DNA until the DNA damage is repaired [41,53]. TIMP-2 and IGFBP7 have been validated for predicting moderate and severe AKI (stages 2–3) in critically ill patients within 12 h of specimen collection [53]. An early (4 h) postoperative increase in the two biomarkers in high-risk cardiac surgery patients has a high predictive value for AKI development [54]. Multiplying the concentrations of both biomarkers (i.e., [TIMP-2]•[IGFBP7]) yields an AKI risk score, with a score >0.3 (ng/ml)^2^/1000 having 92% sensitivity and 46% specificity while a score >2.0 (ng/ml)^2^/1000 has 37% sensitivity and 95% specificity for the occurrence of AKI stage 2–3 [55]. The reference interval for [TIMP-2]•[IGFBP7] in healthy humans is between 0.04 and 2.22 (ng/ml)^2^/1000. The urine concentration of [TIMP-2]•[IGFBP7] is not increased in stable CKD [56]. [TIMP-2]•[IGFBP7] have been incorporated into the first diagnostic test for AKI approved by the US Food and Drug Administration (FDA)—NephroCheck (Astute Medical, San Diego, CA, USA). The test is also available in many countries worldwide.

Two trials evaluated serial measurements of urinary [TIMP-2]•[IGFBP7]. The ProCESS (Protocolized Care for Early Septic Shock) trial randomly assigned 1351 patients from 31 hospitals presenting with septic shock at the emergency department to receive one of three different resuscitation strategies [57]. Of those patients, 688 had urine [TIMP-2]•[IGFBP7] measurements at enrollment and after 6 h. The development of AKI stage 3, need for kidney replacement therapy (KRT), or death within the first seven days after randomization was evaluated in a secondary analysis of the trial [58]: patients with [TIMP-2]•[IGFBP7] levels >0.3 (ng/ml)^2^/1000 even without clinical evidence of AKI within the first 6 h were more than twice as likely to develop the endpoint compared with those who presented with normal concentrations of the biomarkers**.** Another study involving 530 patients evaluated the utility of serial measurements of [TIMP-2]•[IGFBP7] for predicting AKI stage 2–3: the biomarkers were measured every 12 h during the first 72 h after ICU admission. In patients with three consecutive negative [i.e., ≤0.3 (ng/ml)^2^/1000] tests, the occurrence of AKI stage 2–3 during the first seven days was 13% [59]. In contrast, AKI was present in 57%, 75%, and 94% of patients presenting one, two, or three strongly positive values [i.e., >2.0 (ng/ml)^2^/1000], respectively [59].

A retrospective single-center study evaluated the association of urinary [TIMP-2]•[IGFBP7] with serum procalcitonin and the development of AKI or death**.** These biomarkers were assessed when patients were admitted to a multidisciplinary ICU. Among septic patients, the positivity of both tests implied a higher risk of AKI and death. Furthermore, in patients without sepsis, the double positivity correlated only with higher AKI risk but not mortality [60]. The relevance of these biomarkers was also explored during the first wave of the 2020 pandemic. In a study involving 23 patients hospitalized with coronavirus disease 2019 (COVID-19), the urinary concentrations of urinary [TIMP-2]•[IGFBP7] did not predict AKI development [61]. However, it had notable utility for evaluating AKI progression. Patients who progressed from stage 1 to worse stages had higher concentrations than those who remained in the same stage [61]. None of the AKI patients with [TIMP-2]•[IGFBP7] ≤ 0.3 (ng/ml)^2^/1000 progressed from their initial AKI stage. Conversely, all patients presenting AKI and [TIMP-2]•[IGFBP7] > 2.0 (ng/ml)^2^/1000 progressed to AKI requiring KRT (stage 3D).

### 5.2 Neutrophil gelatinase-associated lipocalin

NGAL is one of the most extensively investigated kidney biomarkers. It is a molecule that binds small iron-carrying molecules called siderophores that act as chelators/transporters in several diseases [62]. NGAL is also involved in tubular epithelial genesis, as it forms an iron-siderophore complex (holo-NGAL), which is secreted by the ureteric bud and can induce tubular epithelial genesis [63]. NGAL also has anti-inflammatory and anti-apoptotic effects [64]. Intravenously administered purified NGAL is taken up in the renal proximal tubules and conserves histologic integrity, function, and cell viability in the proximal tubules after ischemic insult [65]. Notably, the renal protective effects of NGAL are at least in part dependent on heme oxygenase enzyme activity [66]. At least three different types of NGAL have been described, 1) a monomeric 25-kDa glycoprotein produced by neutrophils and epithelial tissues, including tubular cells, 2) a homodimeric 45-kDa protein produced by neutrophils, and 3) a heterodimeric 135-kDa protein produced by tubular cells [32].

NGAL can be measured in urine or plasma and proposed as a distal tubule damage biomarker [42] for diagnosing AKI and estimating AKI severity [32]. Nevertheless, its concentrations and discriminative ability may also be influenced by systemic conditions such as sepsis [67] or NGAL originating from non-kidney tissues [68]. Elevated NGAL levels can be measured <4 h after cardiopulmonary bypass, peaking at 4–6 h [69]. In a recent meta-analysis of more than 13,000 patients [70], the discriminative abilities (area under the receiver operating characteristic curves [AUROCs]) of urinary NGAL were 0.75 (95% confidence interval [CI], 0.73–0.76) and 0.80 (95% CI, 0.79–0.81) for AKI stage 3 and AKI requiring KRT, respectively. For plasma NGAL, the corresponding AUROCs were 0.80 (95% CI, 0.79–0.81) and 0.86 (95% CI, 0.84–0.86), respectively. Cutoff concentrations at 95% specificity for urinary NGAL were >580 ng/ml with 27% sensitivity for AKI stage 3 and > 589 ng/ml with 24% sensitivity for AKI requiring KRT. The corresponding cutoffs for plasma NGAL were >364 ng/ml with 44% sensitivity and >546 ng/ml with 26% sensitivity, respectively [70]. NGAL can be widely measured on clinical laboratory platforms [71].

The BICARBONATE trial, a multicenter, parallel randomized controlled trial comparing perioperative bicarbonate infusion for preventing AKI to usual patient care, explored whether urinary NGAL and the urinary NGAL/hepcidin-25 ratio could predict AKI stage 3D in patients undergoing cardiac surgery with cardiopulmonary bypass [72]. The samples were collected within 1 h after the end of surgery. The rationale for analyzing hepcidin-25 was based on findings demonstrating the concentrations of this solute in patients without AKI [73]. Therefore, it is expected that insults leading to kidney damage would likely increase NGAL and reduce hepcidin-25 concentrations. The analysis included 198 patients, of whom 13 (6.6%) developed AKI stage 3D [72]. The urinary NGAL/hepcidin-25 ratio outperformed urinary NGAL for predicting AKI requiring hemodialysis and in-hospital mortality [72]. Setting the cutoff value for a ratio ≥0.3 established a specificity of 85%. On average, patients requiring KRT had urinary NGAL levels 17 times higher and urinary hepcidin-25 reduced by around 70% compared to people with no AKI. It should be noted that the preoperative NGAL/hepcidin-25 ratio was higher in patients who subsequently developed AKI stage 3D [72].

Considering the newly proposed framework of AKI that considers kidney damage biomarkers in addition to the traditional parameters of kidney function [32], the authors of the BICARBONATE trial reassessed the data from 198 participants and allocated the patients to four groups (Fig. 2) [74]. One hundred and seventy-seven patients (61.6%) presented with no AKI (KDIGO AKI criteria-negative/biomarker-positive), 13 (6.6%) had subfunctional AKI (KDIGO AKI criteria-negative/biomarker-positive), 40 (20.2%) had functional AKI (KDIGO AKI criteria-positive/biomarker-negative), and 18 (9.1%) had combined AKI (KDIGO AKI criteria-positive/biomarker-positive) [74]. Interestingly, subclinical and combined AKI were associated with greater in-hospital mortality than no AKI and clinical AKI (adjusted odds ratio, 28.12, 95% CI 1.47–539.70, p = 0.027; adjusted odds ratio, 3.74, 95% CI 1.75–8.00, p = 0.001) [74]. These findings corroborate the concept that the outcome is worse whenever reduced kidney function is associated with alterations in kidney damage biomarkers.

Serial assessments of urinary NGAL were carried out in a specific pediatric cohort of 66 patients with diabetic ketoacidosis [75]. AKI was present in half of the patients; the majority (24/35) had stage 2 AKI, and the remaining patients (11/35) had stage 3 AKI. Urinary NGAL was significantly higher at admission in patients with AKI versus no AKI (79.8 ± 27.2 vs. 54.6 ± 22.0 ng/ml, p = 0.0002) [75]. Similarly, this pattern remained at the 24-h assessment (61.4 ± 28.3 vs. 20.2 ± 14.5 ng/ml, p = 0.0003). Fluid therapy reduced urinary NGAL and the rise in its concentration at admission correlated with the AKI stage. In that study [75], persistent AKI was defined as a composite outcome of AKI stage 2–3 sustained for >48 h, the development of AKI or progression from AKI stage 1 to a worse stage, need for KRT, or death. AKI recovery occurred in 83% and 91% of the patients at 24 and 48 h, respectively. Urinary NGAL >88 ng/ml had 66% sensitivity and 76% specificity for predicting persistent AKI.

Another specific scenario where NGAL has been deployed was in patients with COVID-19. A retrospective cohort study showed that AKI occurred in 1835 (46%) of 3993 hospitalized adults [76]. Patients with COVID-19 are at risk of developing kidney injury for many reasons, including dehydration and hypotension, sepsis, dysregulated immune responses, and organotropism [77]. A prospective observational study analyzed blood and urine samples from 444 people presenting COVID-19 pneumonia at hospital admission [78]. Of those patients, 198 did not have AKI on presentation to the emergency department. Patients who subsequently developed AKI within 7 days of admission had higher urinary NGAL levels compared to those who did not (158 ± 237 ng/ml [n = 51] vs. 74 ± 65 ng/ml [n = 147]; p < 0.05). Patients presenting with sustained AKI for >72 h compared to those who did not also had higher urinary NGAL concentrations (332 ± 324 vs. 96 ± 139 pg/ml; p < 0.0001), with a stepwise increase with increasing AKI severity. Moreover, urinary NGAL had 80% specificity and 75% sensitivity for diagnosing AKI stage 2–3 with a cutoff of 150 ng/ml [78]. A small single-center report involving 17 patients with COVID-19 admitted to the ICU found that 59% developed AKI. The mean urinary NGAL was 93 ng/ml in those with AKI compared to 26.6 ng/ml NGAL in those without AKI; the optimal NGAL cutoff value for predicting AKI was identified as 73 ng/ml [79].

### 5.3 Kidney injury molecule 1

Urinary kidney injury molecule 1 (KIM-1) is a 38.7-kDa type 1 transmembrane glycoprotein produced by proximal tubular cells. It is released into the urine in response to tubular damage and functions in clearing apoptotic cells, and has anti-inflammatory properties [43,80,81]. In animal models, KIM-1 has been correlated with the severity of histological tubular injury [82], which is why the FDA approved its use as an AKI biomarker for preclinical drug development [83]. For clinical practice, KIM-1 has been proposed as a biomarker of proximal tubule damage [43] for predicting AKI, diagnosing AKI, and estimating AKI severity [32]. A recent meta-analysis involving adult patients reported that KIM-1 was a good predictor of AKI, with an estimated sensitivity of 0.74 (95% credible interval, 0.62–0.84) and specificity of 0.86 (95% credible interval, 0.76–0.90) [84]. Elevated KIM-1 levels can be measured 12–24 h after tubular injury, peaking at 2–3 days [85]. KIM-1 can be widely measured on clinical laboratory platforms [86].

### 5.4 Liver-type fatty acid-binding protein

Liver-type fatty acid-binding protein (L-FABP) is a 14-kDa intracellular lipid chaperone that mobilizes lipid peroxides from the cytoplasm of tubular epithelial cells to the tubular lumen. L-FABP gene expression is increased by peroxisome proliferator-activated receptor α [87] and hypoxemia [88]. L-FABP is freely filtered by the glomerulus and reabsorbed in the proximal tubule. Therefore, elevated serum and urinary L-FABP levels can indicate proximal tubular cell damage [44,89] and have been proposed as biomarkers for diagnosing AKI [32]. A recent meta-analysis reported that the estimated sensitivity of L-FABP for predicting AKI was 0.75 (95% CI, 0.69–0.80), and specificity was 0.78 (95% CI, 0.71–0.83) [90]. Furthermore, L-FABP demonstrated an AUROC of 0.82 (95% CI, 0.79–0.85) in variable clinical settings, including ICU, surgery, and contrast-induced AKI [90]. In subgroup analysis excluding pediatric and post-radiocontrast exposure cohorts, L-FABP had a comparative diagnostic performance with NGAL [90]. The appearance and time to peak levels of L-FABP following injury are unknown [1].

### 5.5 C–C motif chemokine ligand 14

The RUBY study, an international prospective observational study designed for detecting biomarkers associated with persistent (≥72 h) AKI stage 3, identified urinary C–C motif chemokine ligand 14 (CCL14) as a reliable predictor of persistent AKI [45]. The cohort included exclusively participants in the ICU (not ward) within 36 h of meeting KDIGO AKI stage 2–3 based on serum creatinine or urine output criteria. Approximately one-third of the patients (110 of 331) matched the criteria for persistent KDIGO AKI stage 3, of whom 56 (51%) received KRT. Urinary CCL14 had an AUROC of 0.83 (95% CI, 0.78–0.87) for predicting persistent AKI. Bagshaw and collaborators conducted a secondary analysis of the prospective SAPPHIRE study to validate the RUBY study findings [91]. That analysis involved 195 patients who developed AKI stage 2 or 3, of whom 28 (14%) progressed to or remained at AKI stage 3. Urinary CCL14 showed adequate accuracy with an AUROC of 0.81 (95% CI, 0.72–0.89). Notably, the risk for persistent AKI increased with greater urinary CCL14 values.

### 5.6 Urinary dickkopf-3

Urinary dickkopf-3 is a 38-kDa stress-induced, renal tubular epithelia-derived secreted glycoprotein that induces tubulointerstitial fibrosis through its action on the canonical Wnt–β-catenin signal pathway [47] by promoting renin-angiotensin-aldosterone activation, stress-induced cytokine (e.g., interleukin-6 and interleukin-8) expression in tubular epithelial cells, uncontrolled fibroblast activation, and extracellular matrix deposition [47,92]. Although sustained activation of Wnt–β-catenin signaling is considered detrimental and promotes tubulointerstitial fibrosis, transient activation is thought to mitigate initial injury and accelerate subsequent recovery after AKI [93,94]. Therefore, persistently elevated urinary dickkopf-3 levels may indicate ongoing tubular “stress” leading to progressive kidney fibrosis in various-cause CKD [95,96]. Currently, urinary dickkopf-3 is the only biomarker that has been validated for AKI risk assessment before exposure to kidney injury [32]. In 733 patients undergoing cardiac surgery, the preoperative urinary dickkopf-3/creatinine ratio predicted the development of postoperative AKI and kidney function loss with an AUROC of 0.78 [97]. Furthermore, a preoperative urinary dickkopf-3/creatinine concentration ratio >471 pg/mg was associated with a significantly higher risk of persistent kidney dysfunction (odds ratio, 6.67; 95% CI, 1.67–26.61; p = 0.007) and dialysis dependency (odds ratio, 13.57; 95% CI, 1.50–122.77; p = 0.02) after 90 days compared with a ratio of ≤471 pg/mg [97]. Urinary dickkopf-3 may also have a role in assessing AKI transition to CKD [98]. Urinary dickkopf-3 can be measured using a commercially available enzyme-linked immunosorbent assay (ELISA) platform (ReFiNE; DiaRen UG, Homburg/Saar, Germany) with a lower detection limit of 30 pg/mg [95].

### 5.7 Cystatin C

Cystatin C, an established surrogate marker of GFR, is a cysteine protease inhibitor formed at a relatively constant rate by all nucleated cells [48]. Due to its small molecular size (13.3 kDa) and basic pH, it is freely filtered at the glomerulus, reabsorbed, and catabolized by the proximal tubule cells. Accordingly, the serum cystatin C concentration can be regarded as an indicator of the GFR. However, compared to serum creatinine, cystatin C is not influenced by sex or age and only shows a lower susceptibility to muscle mass and food intake [99]. Important confounders for the cystatin C level are thyroid diseases, inflammation, smoking, obesity, and cortisone therapy [48]. It should be noted that cystatin C is a marker of the GFR and not tubular damage. The use of cystatin C in CKD has been adequately described. The KDIGO guidelines recommend measuring cystatin C in adults with a serum creatinine-based estimated GFR (eGFR) of 45–59 ml/min/1.73 m^2^ to confirm CKD diagnosis, provided no other renal structural abnormalities are present [100]. Cystatin C has been proposed as a biomarker of kidney dysfunction for diagnosing AKI and estimating AKI severity [32]. Cystatin C can be widely measured on clinical laboratory platforms and is assayed using methods that are traceable to International Federation of Clinical Chemistry and Laboratory Medicine (IFCC)- and Institute for Reference Materials and Measurements (IRMM)-certified reference materials [101].

### 5.8 Proenkephalin A

Proenkephalin A is a stable fragment derived from the precursor enkephalin, a small endogenous opioid peptide produced throughout the human body, including the kidneys [102]. The glomeruli freely filter proenkephalin A due to its small molecular size (4.5 kDa); it has no known tubular handling or extrarenal clearance. It is not bound to plasma proteins and is stably produced in various disease states independent of inflammation and other non-renal factors [49]. Therefore, proenkephalin A is considered a marker of kidney function. Its plasma concentrations have been strongly negatively correlated with measured GFR [49,103]. A study of 24 septic patients in the ICU reported that proenkephalin A concentration levels were highly correlated with measured GFR calculated with iohexol (r^2^ = 0.91; p < 0.001) [104]. In patients with chronic heart failure, proenkephalin A was strongly associated with GFR measured with ^131^I-hippuran and ^125^I-iothalamate (standardized beta = −0.71; p < 0.001) [105].

Endogenous opioid peptides also exert cardiodepressant effects [106] and kidney effects predominantly by increasing renal blood flow and urinary output [107]. Enkephalins might play a pathophysiological role in the development and progression of cardiorenal syndrome by their established effects on cardiac contractility, hemodynamics, and kidney function. In line with that, elevated proenkephalin A levels are also observed in patients with heart failure [105,108]. Age and sex are not associated with proenkephalin A concentrations in healthy people (reference interval: 36–97.5 pmol/l) [109]. Proenkephalin A can be measured at the bedside using a clinical immunoassay (IB10 sphingotest®; Nexus-Dx, San Diego, CA, USA). It can also be measured on clinical laboratory platforms using a chemiluminescence immunoassay [110].

## 6 Biomarker panel

A panel of biomarkers can enrich AKI diagnosis, yielding insights from the pathophysiological mechanisms of different AKI subtypes and highlighting the most affected segments of the renal tubules. Simultaneous analysis of a group of biomarkers can yield clues to the predominant etiology of coexisting kidney insults (e.g., sepsis, hypovolemia, nephrotoxicity) [40]. An observational study enrolled 1635 patients at the emergency departments of three hospitals to explore the diagnostic and predictive abilities of five urinary biomarkers (NGAL, interleukin-18, KIM-1, L-FABP, cystatin C). NGAL demonstrated good discriminatory ability for detecting AKI and was related to AKI duration [111].

Using a panel of urinary biomarkers (NGAL, KIM-1, *N*-acetyl-β-glucosaminidase), McWilliams and co-workers confirmed the proximal tubule as the expected anatomical site of injury in the nephrons of premature neonates exposed to aminoglycosides [112]. Forty patients received gentamicin treatment during the first week after birth. Significant elevations in the three biomarkers were observed during the gentamicin treatment courses, returning to baseline values once the drug was discontinued. When adjusted for confounders, the treatment effect of gentamicin remained significant only for KIM-1, a marker of proximal tubule injury [112].

In the context of acute cardiorenal syndromes, 283 hospitalized patients undergoing decongestive therapy for acute decompensated heart failure with furosemide (median, 560 mg/day) presented normal urinary NGAL, KIM-1, and *N*-acetyl-β-glucosaminidase, irrespective of a reduction in their cystatin C-based eGFR [113]. These results demonstrate that during decongestive treatment of heart failure, changes in kidney function markers might be dissociated from kidney injury markers, a condition described as functional AKI (KDIGO AKI criteria-positive/biomarker-negative) [34]. It is worth noting that in a large randomized clinical trial comparing decongestive therapies, i.e., the DOSE (Diuretic Optimization Strategies Evaluation) trial [114], increments in serum creatinine >0.3 mg/dl (i.e., KDIGO AKI stage 1) were not associated with negative outcomes, probably reflecting only glomerular hemodynamic changes without tubule cell damage. Therefore, the authors stated that using mild-to moderate-sized changes in serum creatinine as an endpoint in heart failure clinical trials may be inappropriate [115]. Moreover, the nephrotoxicity of iodinated intravenous contrast was investigated using a panel of biomarkers. NGAL, KIM-1, and interleukin-18 concentrations were measured in 922 participants at baseline and 2–4 h after contrast injection and demonstrated no changes, even in patients who developed KDIGO AKI [116]. These findings suggest that most cases of contrast-associated AKI likely reflect functional AKI (KDIGO AKI criteria-positive/biomarker-negative) rather than intrinsic kidney damage [116].

The DAMAGE (Dublin Acute Biomarker Group Evaluation) study was a prospective observational trial investigating the utility of urinary AKI biomarkers on ICU admission at two academic medical centers. The study recruited 717 patients. The primary outcome was developing any stage of AKI during the first seven days following ICU admission. Only the KDIGO serum creatinine criteria but not the urine output criteria were used in the AKI staging. The urinary panel consisted of NGAL, albumin, KIM-1, L-FABP, and cystatin C. Thirty-eight percent of the patients developed AKI, 215 (84%) during the first 48 h and 42 (16%) after 48 h but before seven days [117]. A secondary analysis of the DAMAGE study assessed 14 urinary biomarkers [118]. In the group of patients that presented AKI stage 1 or 2 within 48 h after ICU admission, eight biomarkers were associated with progression to worse AKI stages, KRT, or death in 7 days. The best predictors for these outcomes were cystatin C, interleukin-18, albumin, and NGAL. Evaluation of the patients who developed AKI within the first seven days after enrollment showed that interleukin-18, NGAL, albumin, and monocyte chemotactic protein-1 were independently associated with KRT or death within 30 days.

## 7 Cost-effectiveness of using biomarkers to detect AKI

Data indicate that AKI is associated with 13%–18% of hospital admissions [119] and affects up to 50% of critically ill patients [120]. Patients who develop AKI in hospital are also more likely to have longer length of stay and higher odds of in-hospital death [121]. As a consequence, the association of AKI with higher morbidity and mortality is also linked to increased costs that range from $5.4 USD to $24.0 USD billion annually in the U.S [122]. The use of novel AKI biomarkers may aid hospital systems in implementing management strategies, such as care bundles [36,37] to prevent AKI or its progression to a higher stage, thereby improving outcomes and reducing healthcare costs. Although cost-effectiveness evidence for different biomarker tests for early detection of AKI is sparse [[123], [124], [125]], existing evidence suggests that these may be cost effective when considered along with the standard of care (i.e., serum creatinine). Notably, the higher the prevalence of AKI, the more likely the tests are to be cost-effective. This implies that careful consideration should be given to identifying subsets (e.g. post-major surgery) of those in hospital who would be most likely to benefit from testing and could be targeted in future trials.

## 8 Future research

Although several markers have been shown to detect AKI earlier and are more sensitive than the current markers of AKI (i.e., serum creatinine and urine output), there are still substantial knowledge gaps that need to be covered in future studies. A recent consensus paper on AKI biomarkers made recommendations for clinical practice and future research [32]. In particular, there is a need for interventional studies evaluating the utility of biomarker measurement in guiding treatment strategies to prevent progression of AKI. Currently, only limited data is available to support an impact of a positive biomarker test guided implementation of an AKI care bundle in high-risk patients [36,37,126]. Considering the limitations, the results from ongoing multicentre studies are required to generate more evidence (e.g., BigpAK-2 [Biomarker-guided Intervention to Prevent Acute Kidney Injury; NCT04647396], LAPIS [A Study Looking at the Use of Biomarkers to Provide Early Indication of Acute Kidney Injury in Patients With Sepsis (Limiting AKI Progression In Sepsis); NCT04434209]). It is hoped that the data provided from these studies, among others, will also be a valuable resource from which to inform future economic evaluations.

Finally, laboratory implementation of AKI biomarkers requires the determination of reference intervals, their widespread availability, and concordance between assays. For example, a recent position paper of the American Association for Clinical Chemistry highlighted that the cystatin C assay cannot be universally recommended due to poor standardization, the lack of availability from most vendors and high cost (in comparison with serum creatinine) worldwide [127]. We anticipate that cost reduction of cystatin C measurements could occur with broad adoption over time given that the National Kidney Foundation-American Society of Nephrology recommends that clinical laboratories quickly implement the CKD Epidemiology Collaboration (CKD-EPI) 2021 equations based on serum creatinine and cystatin C to eliminate race as a parameter when calculating estimated GFR and to standardize laboratory reporting [128]. Beyond cystatin C, the only other biomarker approved by the FDA is [TIMP-2]•[IGFBP7] for the assessment of AKI stage 2–3; it is also available in Europe. NGAL has also been CE-Marked (Conformité Européenne) since 2009 but is has not been approved by FDA for clinical use.

## 9 Conclusions

The recent gain of knowledge in the field of AKI reflects the advances in molecular biology. The role of several molecules involved in complex AKI mechanisms has been partially unraveled, propelling the investigation of the clinical utility of these molecules as biomarkers. In the roadmap for an update in AKI classification the incorporation of biomarkers is of utmost relevance. The judicious use of the new clinical available biomarkers allows the early laboratory detection of AKI and the recognition of AKI subphenotypes. This is especially pertinent in high-risk patients in whom the additional information brought by laboratory results enriches and makes risk assessment tools more reliable. Preventive actions and early interventions can be tailored to the patients that might benefit them most, ultimately mitigating the burden and adverse outcomes associated with AKI.

A wake-up call is needed to alert healthcare policymakers and clinical practitioners to integrate the new biomarkers in protocols for AKI management. In parallel, researchers are encouraged to assess biomarkers as outcomes in trials evaluating their cost-effectiveness and the safety profile of pharmacological and non-pharmacological interventions concerning the kidneys. Undoubtedly, these actions will be major drivers in the progress of nephrology.

## Funding

This work did not receive any specific grant from funding agencies in the public, commercial, or not-for-profit sectors.

## Declaration of competing interest

TR has received funding for lectures, been consultant or advisory board member for AstraZeneca, B. Braun, Baxter, bioMérieux, Contatti Medical (CytoSorbents), Eurofarma, Jafron. CR has received funding for lectures, been consultant or advisory board member for Asahi, Astute, B. Braun, Baxter, bioMérieux, Bioporto, CytoSorbents, Estor, Fresenius Medical Care, General Electric (GE), Jafron, Medtronic, Toray. None of the other authors declare any competing interests. The authors shared manuscript design, literature collection, literature analysis and interpretation, preparation, review, and approval of the manuscript. The corresponding author had full access to all of the work and had final responsibility for the decision to submit for publication.

## Abbreviations

AKI: acute kidney injury
AUROC: area under the receiver operating characteristic curve
CCL14: C–C motif chemokine ligand-14
CKD: chronic kidney disease
FDA: US Food and Drug Administration
GFR: glomerular filtration rate
ICU: intensive care unit
IGFBP7: insulin-like growth factor binding protein
KDIGO: Kidney Disease: Improving Global Outcomes
KIM: kidney injury molecule-1
KRT: kidney replacement therapy
NGAL: neutrophil gelatinase-associated lipocalin
TIMP-2: tissue inhibitor of metalloproteinases-2
